# Is thirst the missing physiological link between SGLT2 inhibitors and reduced nephrolithiasis risk?

**DOI:** 10.1093/ckj/sfag133

**Published:** 2026-04-29

**Authors:** Marco Lombardi, Paolo Beltrami, Bernardo Martini

**Affiliations:** CAREX – Italian Association of Kidney Stone Patients (APS), Florence, Italy; CAREX – Italian Association of Kidney Stone Patients (APS), Florence, Italy; CAREX – Italian Association of Kidney Stone Patients (APS), Florence, Italy

To the Editor,

For decades, increasing fluid intake has been the cornerstone of nephrolithiasis prevention. Patients with kidney stones are routinely advised to increase daily water consumption in order to achieve a urinary output exceeding 2–2.5 l/day. Despite its central importance, long-term adherence to hydration strategies remains highly variable among patients, suggesting that biological determinants of thirst may play an important role in shaping fluid intake behavior. However, the physiological regulation of thirst has been relatively underexplored in nephrology, where hydration is often considered primarily as a behavioral intervention rather than a biologically regulated variable.

Thirst and vasopressin represent two coordinated outputs of the central osmoregulatory system. Osmoreceptors located in the circumventricular organs—particularly the subfornical organ and the organum vasculosum of the lamina terminalis—detect subtle changes in plasma osmolality and circulating angiotensin II. These signals are integrated within hypothalamic nuclei that simultaneously stimulate thirst perception and arginine vasopressin secretion. Vasopressin acts on V2 receptors in the collecting duct, promoting aquaporin-2 insertion and renal water reabsorption, thereby concentrating the urine. Copeptin, the stable C-terminal fragment of the vasopressin precursor, is considered a surrogate marker of vasopressin secretion in clinical research settings [1].

**Figure 1:**
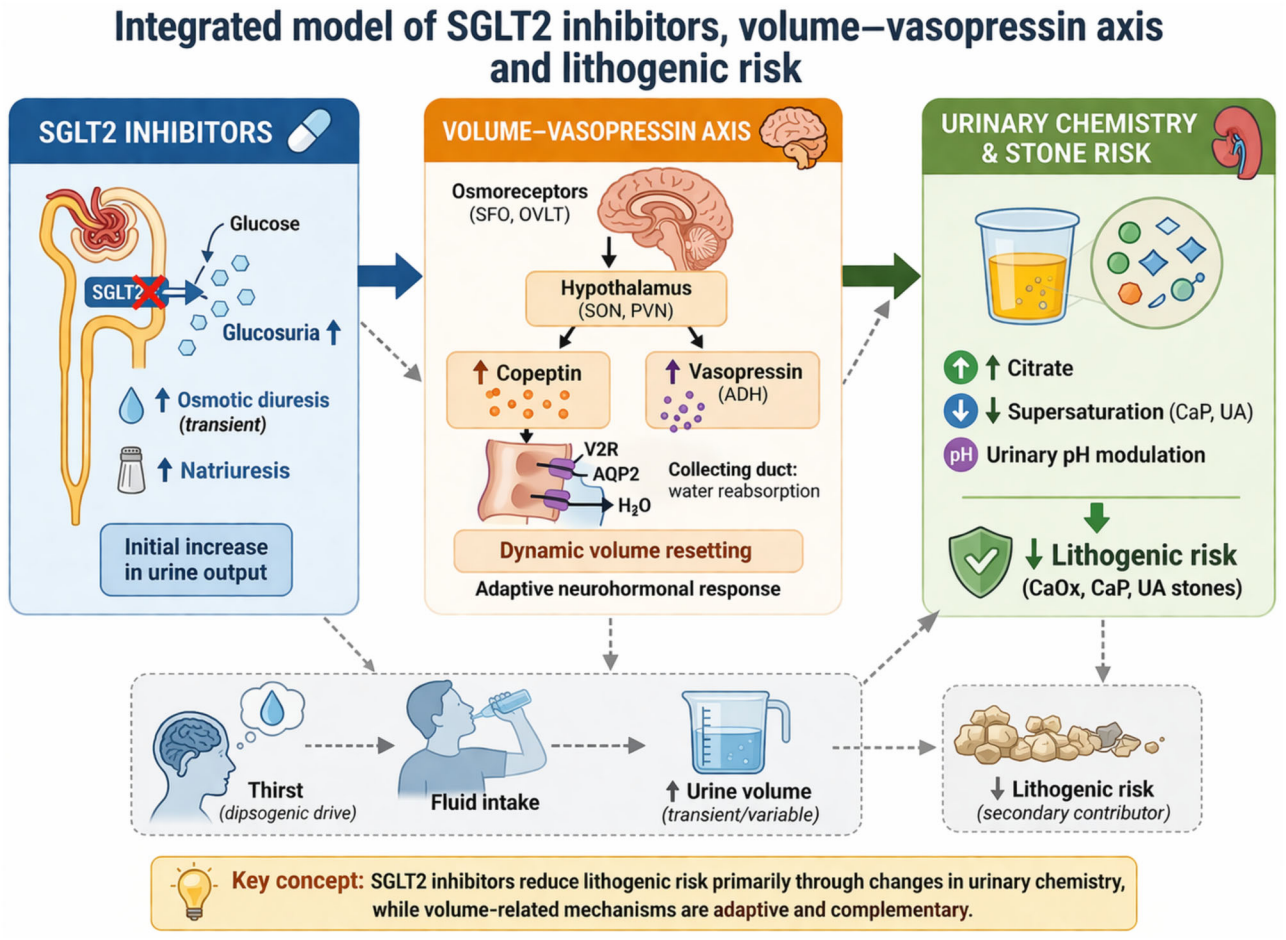
Integrated model of SGLT2 inhibitors, volume–vasopressin axis and lithogenic risk. SGLT2 inhibitors induce glucosuria, transient osmotic diuresis, and natriuresis. These changes trigger adaptive neurohormonal responses, including activation of the vasopressin–copeptin axis and dynamic resetting of volume homeostasis. While osmotic diuresis may transiently influence thirst and fluid intake, current evidence suggests that sustained increases in urine volume are not the primary mechanism underlying the antilithogenic effects of SGLT2 inhibitors. Instead, these agents reduce lithogenic risk predominantly through changes in urinary chemistry, including increased citrate excretion, modulation of urinary pH, and reduced supersaturation of calcium phosphate and uric acid. Volume-related mechanisms are therefore likely complementary rather than causal.

From a lithogenic perspective, this pathway can be conceptualized as a physiological continuum in which increased vasopressin activity promotes higher urine osmolality and lower urinary volume, thereby increasing urinary supersaturation of lithogenic solutes such as calcium oxalate, calcium phosphate, and uric acid (Fig. 1). In this context, vasopressin may act as a physiological amplifier of lithogenic risk, promoting sustained urine concentration and increased supersaturation. Consistent with this concept, pharmacological blockade of vasopressin V2 receptors with tolvaptan has been shown to increase urine volume and reduce urinary supersaturation in recurrent stone formers [2].

We propose that a subset of patients with nephrolithiasis may exhibit a putative “neuro-osmotic lithogenic phenotype,” characterized by relatively increased vasopressin activity and persistently concentrated urine. However, this concept should be regarded as hypothesis-generating and is not currently supported by direct disease-specific evidence. In this context, copeptin may represent a potential research biomarker, although its role in nephrolithiasis remains to be established.

Recent pharmacological observations suggest that thirst may be a modifiable neuroendocrine output. In a reported case, treatment with a glucagon-like peptide-1 receptor agonist (liraglutide) reduced severe thirst and interdialytic weight gain in a patient undergoing chronic hemodialysis [3]. While not directly related to nephrolithiasis, this observation supports the concept that central regulation of thirst can be pharmacologically influenced.

These considerations provide a framework to reconsider the mechanisms underlying the association between sodium–glucose cotransporter-2 (SGLT2) inhibitors and nephrolithiasis. Large observational studies have reported a lower incidence of kidney stones among patients treated with SGLT2 inhibitors compared with other glucose-lowering therapies [4].

Recent randomized evidence has provided important mechanistic insights. In the SWEETSTONE trial, short-term SGLT2 inhibition improved urinary supersaturation in nondiabetic kidney stone formers without a significant increase in urine volume, indicating that changes in urinary chemistry—rather than sustained increases in fluid intake—are likely the dominant drivers of the antilithogenic effect [5].

Accordingly, the effects of SGLT2 inhibitors on fluid balance appear to be complex and time-dependent. Osmotic diuresis is typically transient and followed by compensatory mechanisms, including vasopressin activation and normalization of fluid balance. Importantly, several experimental and clinical studies have demonstrated that SGLT2 inhibitors induce adaptive renal and neurohormonal responses related to volume homeostasis, including increases in copeptin, natriuresis, and activation of volume-regulatory pathways [6–11].

These findings suggest that SGLT2 inhibitors do not induce sustained urinary dilution, but rather a dynamic resetting of the volume–vasopressin axis rather than sustained urinary dilution.

Accordingly, any contribution of thirst-driven fluid intake to the antilithogenic effects of SGLT2 inhibitors should be considered exploratory and complementary rather than causal.

Nevertheless, osmotic diuresis induced by glucosuria may still interact with central dipsogenic pathways, potentially influencing fluid intake in selected individuals (Fig. 1). In this context, thirst may represent one component of a broader integrative physiological response linking systemic osmoregulation to urinary chemistry rather than a primary mechanistic driver.

Recognizing thirst as a physiological variable rather than solely a behavioral factor may open new perspectives in the pathophysiology of kidney stone disease. Whether modulation of the thirst–vasopressin axis contributes to nephrolithiasis risk, or interacts with the effects of SGLT2 inhibitors, remains an open and testable hypothesis that warrants dedicated prospective investigation.
